# Impact of somatic and addictive comorbidities on the quality of life of patients with schizoaffective disorder

**DOI:** 10.1192/j.eurpsy.2023.1233

**Published:** 2023-07-19

**Authors:** W. Bouali, N. Faouel, R. Ben Soussia, F. Zaouali, L. Zarrouk

**Affiliations:** Psychiatrie, Faculty of Medicine of Monastir, Mahdia, Tunisia

## Abstract

**Introduction:**

Schizoaffective Disorder is frequently associated with somatic and addictive comorbidities. This association can change the expression of the disease as well as its prognosis. In addition, this association can affect many functional and psychosocial aspects that can lead to impaired quality of life (QOL) and overall functioning of patients.

**Objectives:**

the aims of this work were to study the impact of somatic and addictive comorbidities on QOL and global functioning of patients followed for schizoaffective disorder.

**Methods:**

This is a cross-sectional study carried out at the psychiatric consultation of the University Hospital of Mahdia for a period of 6 months. The evaluation of the QOL was made using a generic instrument for measuring quality of life: the SF-36 in its version in literary Arabic using the Global Evaluation of Functioning scale .

**Results:**

fifty-two patients with schizoaffective disorder were included in the study. The age of the patients varied from 29 to 62 years with an average of 38 years. The sex ratio (M/F) was 1.6. Singles accounted for 46.2%. Somatic comorbidities were found in 30.8% of patients. Diabetes ranked first (13.5%) followed by arterial hypertension (9.6%) then epilepsy (3.8%). Addictive comorbidities were noted in 63.5% of patients. Tobacco, alcohol and Cannabis were the most consumed substances with respective rates of 57.7; 28.8 and 13.5%. The evaluation of the QoL of the patients revealed that 80.8% of the patients had scores attesting to an altered QoL. Regarding the evaluation of global functioning by EGF, (65.4%) of patients had a score of less than 70 attesting to an impairment of global functioning. The analytical study of correlation between the dimensions of the SF-36 and somatic comorbidities found that dimension D1 (physical activity) is significantly influenced by somatic comorbidities (10-4). The deterioration in the global functioning of the patients was not correlated with the presence of somatic comorbidities (p=0.28). The change in QoL was not correlated with the consumption of psychoactive substances (0.32 for alcohol and p=0.23 for drug addiction).

**Conclusions:**

It is accepted that the objectives of the management of patients suffering from schizoaffective disorder go beyond the remission of clinical symptoms to the improvement of QoL and socio-professional functioning. Larger-scale work is needed to study the influence of comorbidities associated with schizoaffective disorder on these dimensions.

**Disclosure of Interest:**

None Declared

